# Plant-Derived Diamine Oxidase Modulation of Histamine-Induced Ca^2+^ Release in Intestinal Caco-2 Cells: A Cellular System to Evaluate Its Histaminase Efficacy

**DOI:** 10.3390/cells15131175

**Published:** 2026-06-28

**Authors:** Armelle Tchoumi Neree, Catherine Jumarie, Lucia Marcocci, Paola Pietrangeli, Mircea Alexandru Mateescu

**Affiliations:** 1 Department of Chemistry, University of Quebec at Montreal (UQAM), Montreal, QC H3C 3P8, Canada; tchoumi_neree.armelle@courrier.uqam.ca; 2 Centre Protéo—UQAM, Montreal, QC H3C 3P8, Canada; 3 Groupe de Recherche TOXEN—UQAM, Department of Biological Sciences, UQAM, Montreal, QC H3C 3P8, Canada; 4 Department of Biochemical Sciences “A. Rossi Fanelli”, “Sapienza” University of Rome 1, 00185 Rome, Italy; lucia.marcocci@uniroma1.it (L.M.); paola.pietrangeli@uniroma1.it (P.P.)

**Keywords:** calcium (Ca^2+^) mobilization, histamine, H1 Receptors (H1Rs), vegetal diamine oxidase, histaminase, desloratadine (Des), fluorescent Ca^2+^ indicator Fluo-4, histamine intolerance, intestinal epithelial cells Caco-2

## Abstract

**Highlights:**

- Histamine induces Ca^2+^ release in enterocyte-like Caco-2 cells via H1 receptors (H1R).
- Histamine Ca^2+^ stimulation is fast (less than 50 s), cumulative and repetitive (after cell washings).
- Diamine oxidase (DAO) disruption of histamine reduces H1R–mediated Ca^2+^ signaling.
- Vegetal (vDAO) exerts a comparable inhibition of Ca^2+^ release as desloratadine, but at a lower concentration.
- Oral forms of vDAO may alleviate some intestinal histamine-related dysfunctions

**Abstract:**

Histamine accumulation is associated with allergic reactions and intestinal dysfunction through activation of H1R Receptors (H1Rs) and subsequent intracellular Ca^2+^ mobilization. Although antihistamines such as desloratadine block H1Rs to alleviate symptoms, they may induce adverse effects (dizziness, nausea, neurological disorders). Diamine oxidases (DAOs) catalyze the oxidative deamination of histamine to imidazole acetaldehyde with consumption of oxygen and concomitant production of hydrogen peroxide and ammonia. Instead of blocking H1R, we have proposed vegetal diamine oxidase (vDAO) as an enzymatic approach to decompose histamine. This study investigated whether vDAO, by degrading histamine, could modulate histamine-induced Ca^2+^ mobilization compared with desloratadine. Using intestinal Caco-2 cultured cells loaded with the fluorescent Ca^2+^ indicator Fluo-4 AM, intracellular calcium (Ca^2+^) dynamics were analyzed by confocal microscopy following histamine stimulation. vDAO significantly downregulated histamine-induced Ca^2+^ mobilization. Notably, at a fixed histamine concentration sufficient to trigger Ca^2+^ release, vDAO achieved the same level of calcium mobility inhibition at a concentration three-fold lower than that of desloratadine, suggesting a high efficiency of DAO. These findings indicate that histamine degradation with orally administrable DAO may reduce Ca^2+^ signaling in intestinal epithelial cells, providing a promising approach to reduce histamine-related damage or a reinforcement of current antihistamine anti-inflammatory medication based on histamine receptor blockade.

## 1. Introduction

Histamine is a biogenic amine, endogenously generated by decarboxylation of his-tidine by histidine decarboxylase in various eukaryotic cells (i.e., mastocytes, basophils, gastric enterochromaffin-like cells, neurons) and in some bacteria of the intestinal microbiota [1,2,3,4]. Histamine is also exogenously provided by the intake of fermented food and beverage [5,6,7]. This aromatic amine is involved in many biological processes including the control of gastrointestinal secretion [8], inflammation, allergic and pseudo-allergic reactions [9,10], neurotransmission [11], intestinal cell proliferation, migration and differentiation [12,13] and gut contractibility [14]. Specifically, higher levels of histamine have been associated with gastrointestinal (GI) dysfunctions (Crohn’s disease, ulcerative colitis, irritable bowel syndrome, histamine intolerance), neurological impairment (cognition, sleep–wake cycle, nociception), and tumor development and progression [8,15,16,17,18]. The effects of histamine are mediated by histamine receptors H1 to H4 which activate cellular G protein signaling pathways [19,20]. H1Rs, of interest in this study, are expressed by a broad range of mammalian cell types including enterocytes [21,22].

The enterocyte-like Caco-2 cells isolated from human colon carcinoma are frequently used as an in vitro model mimicking human gut properties [23,24] in investigation of intestinal epithelium features.

**Scheme 1:**
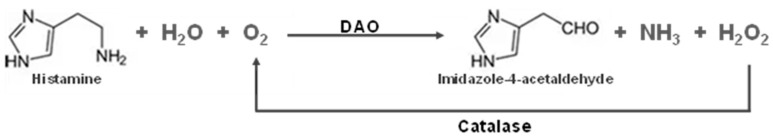
Oxidative decomposition of histamine catalyzed by DAO and disruption of H_2_O_2_ catalyzed by Catalase.

Diamine oxidase (DAO), also known as histaminase (EC 1.4.3.6), is an oxidoreductase that catalyzes the oxidative deamination of primary amino groups in certain biogenic amines such as histamine, tyramine, putrescine, and cadaverine [25,26,27] yielding the corresponding aldehydes along with production of hydrogen peroxide and ammonia (Scheme 1).

In the intestinal lumen, an endogenous DAO is released from enterocytes [14,28] and may be reinforced or completed with orally administered food supplements as tablet forms of DAO from pig kidney [29,30,31] or from plants such as *Lathyrus sativus* or *Pisum sativum* [32,33]. The endogenous intestinal DAO may be inhibited in certain individuals due to an intestinal dysbiosis that could damage the intestinal mucosa where DAO is produced [34]. A low activity of intestinal DAO may also be due to genetic factors such as nucleotide polymorphisms of the AOC1 gene that encodes the DAO enzyme [35]. Furthermore, several types of drugs such as chloroquine, clavulanic acid, cimetidine, verapamil are known to generate 50% (or more) inhibition of DAO [36]. Also, metformin, the most prescribed and consumed oral anti-diabetic drug, inhibits DAO in the intestinal tract [37]. In rare conditions, food histamine may generate scombroid poisoning, anaphylaxis and even death [38]. This histamine intoxication is not an allergy but rather a so-called “pseudo allergy”, a histamine intolerance (HIT) often due to diamine oxidase (DAO) deficiency [27]. For all these conditions and medications, orally administered plant-derived DAO may be beneficial to control and prevent histamine-related dysfunctions.

Histamine dysfunctions may be related to an inhibition of endogenous DAO or to common intoxication with histamine from food (frequently fermented food such as sauerkraut or pickles, or food with high histamine levels such as some wines or cheeses). This may trigger symptoms similar to allergic phenomena (pseudo-allergies) or even major allergic conditions, including anaphylaxis [39,40]. Unbalanced histamine levels are quite frequent in food poisoning in allergic phenomena or in cases of histamine-related diseases (such as mastocytosis), which emphasizes the importance of administration of antihistamine drugs or/and of DAO supplements (particularly for intestinal dysfunctions). In recent decades, the intake of oral forms (capsules, tablets) containing DAO purified from pig kidney has been reported to mitigate symptoms in patients affected by food histamine intolerance [12,27,41,42].

Vegetal DAO (vDAO), with an activity several times higher than that from pig kidney, was proposed as an additional tool to alleviate intestinal histamine-related dysfunctions [43]. The efficacy of vDAO to countect the histamine effect was showed in various in vitro cellular systems [13,14,27].

This report aims to investigate the efficiency of DAO to inhibit the histamine-triggered intracellular Ca^2+^ mobility and to compare its efficiency to that of the currently used antihistamine drug desloratadine (Des).

We have previously reported the capacity of vDAO to decrease the histamine-triggered colon contractions on an ex vivo murine model [14] and we have now investigated mechanistic aspects of vDAO controlling the histamine-induced Ca^2+^ mobility on intestinal Caco-2 cells, in comparison with the antihistamine drug Des. The control of Ca^2+^ mobility in epithelial enterocytes is important because they can modulate the release of calcium into the circulation and also from the epithelium to the lumen. Since Ca^2+^ mobility is enhanced by high histamine level, it was of interest to investigate the capacity of DAO (Histaminase) to limit the histamine-induced Ca^2+^ mobility and related intestinal motility that can be painful, particularly in inflammatory disorders.

## 2. Materials and Methods

### 2.1. Materials

Aminoantipyrine (AAP); bovine serum albumin (BSA); calcium chloride (CaCl_2_); ethylenediaminetetraacetic acid (EDTA) disodium salt; sodium 3,5-dichloro-2-hydroxybenzenesulfonate (DCHBS); 1,4-diaminobutane dihydrochloride (putrescine); dibasic sodium phosphate salt (Na_2_HPO_4_) dihydrate; glutamine; 4-(2-hydroxyethyl)-1-piperazine-ethanesulfonic acid (HEPES); histamine; horseradish peroxidase (HRP) type I; monobasic sodium phosphate salt (NaH_2_PO_4_) dihydrate; and probenecid were purchased from MilliporeSigma (Oakville, ON, Canada). Fluo-4 acetoxymethylester (Fluo-4 AM) and pluronic acid were purchased from Thermo Fisher Scientific (Mainway, Burlington, ON, Canada). Dulbecco’s modified eagle medium (DMEM) containing 25 mM glucose, 1× non-essential amino acids and 10,000 U/mL penicillin–streptomycin were purchased from Hyclone (Logan, UT, USA); fetal bovine serum (FBS) was from Wisent Bioproducts (St-Bruno, QC, Canada). The colorectal adenocarcinoma (Caco-2) cell line was sourced from Dr. A Zweibaum [44]. All current chemicals were reagent grade and were used without further purification.

### 2.2. Vegetal Diamine Oxidase Extraction, Purification and Characterization

Diamine oxidase (DAO) was purified from etiolated *Lathyrus sativus* seedlings as reported by [14], previously stored at −80 °C. All procedures were performed at 4 °C to preserve enzyme activity. Frozen seedlings (around 500 g fresh weight) were gently stirred in an equal volume (*w*/*v*) of extraction buffer consisting of 50 mM sodium phosphate buffer (pH 5.5) and 0.2 M NaCl and then filtered through cheesecloth and the filtrate was centrifuged at 15,000× *g* for 30 min at 4 °C. The supernatant (around 200 mL) was collected as the crude enzyme extract that was loaded onto an ion exchange column (YMC-BioPro S75) previously equilibrated with 50 mM sodium phosphate buffer (pH 5.5). Chromatography was performed at a flow rate of 1 mL/min. Fractions (25 mL) were collected and monitored by absorbance at 280 nm. Fractions exhibiting diamine oxidase activity were identified by hydrogen peroxide production during putrescine oxidation using a Lambda 750 spectrophotometer (Perkin Elmer, Shelton, CT, USA). DAO activity was assayed at 25 °C in 0.1 M sodium phosphate buffer with pH 7.2 by following the formation of a pink product (ε_515nm_ = 26,000 M^−1^cm^−1^) generated from the oxidation of 1 mM DCHBS by hydrogen peroxide (H_2_O_2_), produced from the oxidation of 3 mM putrescine, in the presence of 10 μg/mL HRP, and subsequent condensation with 0.1 mM AAP [13,45]. One enzyme unit (U) oxidizes one μmole putrescine/min.

Enzyme-containing fractions were pooled and concentrated by ultrafiltration using centrifugal filter units (MerckMillipore, Kirkland, QC, Canada) with a weight cut-off of 10–30 kDa. The purity of the DAO preparation was evaluated by sodium dodecyl sulfate polyacrylamide gel electrophoresis (SDS-PAGE) under reducing conditions [43,45,46,47], DAO activity was measured spectrophotometrically [48], and protein concentration was determined by the Bradford colorimetric method at 595 nm [49] using bovine serum albumin (BSA) as a standard. Following purification, the enzyme preparation was lyophilized. The resulting purified preparation yield was 305 ± 13 mg of dry powder, corresponding to a protein content of 0.38 ± 0.08 mg protein per mg solid and a specific activity of 23.8 ± 3.3 U/mg solid. The lyophilized enzyme was stored at −80 °C.

### 2.3. Caco-2 Cell Culture

Caco-2 cells (passages 207 to 255) were cultured in DMEM supplemented with 25 mM glucose, 15% heat-inactivated FBS (56 °C, 30 min), 0.1 mM non-essential amino acids, and 50 U/mL penicillin—50 μg/mL streptomycin. Cells were maintained in 75 cm^2^ culture flasks at 37 °C in a humidified atmosphere containing 5% CO_2_. The passage of cells was performed weekly using 0.05% trypsin supplemented with 0.053 mM EDTA, and the culture medium was renewed every two days. For experimental procedures, cells were seeded in 6-well plates at a density of 2 × 10^3^ cells/well and cultured for 21 days to allow differentiation [24,50].

### 2.4. Calcium (Ca^2+^) Flow Measurements

Differentiated Caco-2 cells (21 days) were incubated at 37 °C for 1 h with 1 µM non-fluorescent Fluo-4 acetoxymethyl ester (Fluo-4 AM) calcium (Ca^2+^) indicator, 1µM Pluronic acid and 2.5 mM probenecid, all prepared in 10 mM HEPES buffer (pH 7.2). After incubation, cells were washed three times with HEPES buffer to remove excess extracellular fluorophore. Cells were then incubated with 100 μL of 10 mM HEPES buffer for an additional 30 min to allow intracellular esterases to convert Fluo-4 AM into its active fluorescent form, Fluo-4.

Fluo-4 AM is a membrane-permeant probe able to diffuse passively across the plasma membrane due to its hydrophobic AM ester groups. After entering cells, cytosolic esterases cleave its AM groups, producing Fluo-4 fluorescent indicator. Upon binding intracellular Ca^2+^, Fluo-4 exhibits increased fluorescence intensity, enabling sensitive detection and quantification of dynamic changes in Ca^2+^ concentrations [51,52].

In the current study, the Caco-2 cells were stimulated with histamine. Fluorescence measurements obtained in the absence of vDAO or of desloratadine (Des) are considered as controls. Thus, the resulting intracellular Ca^2+^ mobilization responses enabled a direct comparison of the in vitro effects of vegetal DAO (histaminase) and of Des (antihistamine) in modulating histamine-induced Ca^2+^ signaling.

Confocal microscopy was used to assess the effect of vDAO purified from *Lathyrus sativus* seedlings on histamine-induced Ca^2+^ mobilization in 21-day differentiated Caco-2 cells. To track intracellular calcium release, Caco-2 cells loaded with Fluo-4 AM were exposed to histamine (final concentrations in the range of 0.05–2.50 mM) and vDAO (concentrations in the range of 5–17 μM) or Des (concentrations in the range of 5–20 μM).

Imaging was performed in a humidified 5% CO_2_–air atmosphere at 37 °C, under a Nikon A1 confocal microscope (Nikon corporation, Melville, NY, USA) equipped with a 10× objective. Fluo-4 Ca^2+^ fluorescence was detected at an excitation wavelength (λ_Exc_) of 488 nm and an emission wavelength (λ_Em_) of 525 nm, as previously described [51,52]. Images were acquired in kinetic scan mode for up to 2.5 min, with scans every 2 s, using a pinhole diameter of 11.9 µm, a detector high voltage (HV) of 45, and an offset of −5.

In selected experiments, fluorescence was recorded in real time. Quantitative analysis was performed using Fiji (ImageJ 1.54f software, http://imagej.org, accessed: 10 February 2025). Regions of interest (ROIs) were defined to quantify intracellular fluorescence, and data were expressed as area under the curve (AUC) from time-resolved recordings. Results are presented as mean ± SEM from at least 3 independent experiments. To evaluate inhibitory effects, various concentrations of DAO or Des were co-administered with a fixed concentration of histamine. A washing step was also performed to evaluate the reproducibility of the histamine-induced response.

To better understand the aspects related to histamine triggering Ca^2+^ mobility and the inhibition provided by DAO, the effect of H_2_O_2_ alone on Ca^2+^ mobility was followed as the control. The amounts of H_2_O_2_ generated, in three min, at the oxidation of 1.25 mM histamine with increasing concentrations (5–17 μM) vDAO (same concentrations used to evaluate the Ca^2+^ mobility) were determined by UV/Vis spectrophotometry. Separately H_2_O_2_ alone (no histamine, no vDAO) at same concentrations as those determined at histamine oxidation was added to cultured Caco-2 cells and Ca^2+^ mobility was evaluated with the Fluo-4 AM sonde by confocal microscopy. Enzyme and H_2_O_2_ solutions were prepared in 10 mM HEPES buffer, with pH 7.2.

### 2.5. Statistics

Data are expressed as mean ± SD of at least *n* = 5 different measurements. Statistical tests were performed with the GraphPad Prism 9.5.1, using either one-way ANOVA (for comparison between three groups) or two-tailed Student’s *t*-test) (for comparison between two groups). Statistical significance was defined as *p < 0.1 and **p < 0.01.

## 3. Results and Discussion

The 21-day-old differentiated Caco-2 cells represent an accepted model which correlates well with the permeability barrier function of the intestinal epithelium cells [53,54]. Histamine activation of H1 receptors (H1Rs) regulates the Ca^2+^ intracellular concentration and its pathways in most of the intestinal cells [55].

### 3.1. Histamine-Induced Ca^2+^ Mobility in 21-Day Differentiated Caco-2 Cells

**Figure 1:**
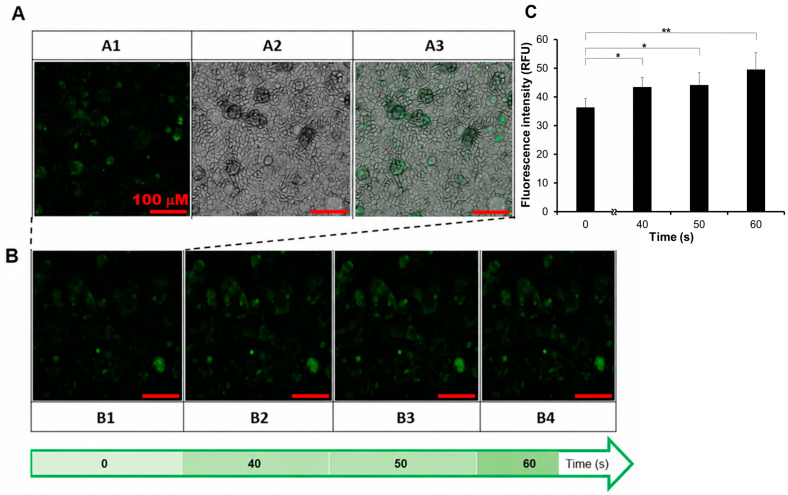
Ca^2+^ mobilization in 21-day differentiated Caco-2 cells loaded with Fluo-4. (**A**): Non-stimulated cells; the green fluorescence (**A1**), transmitted light (**A2**) and (**A1**,**A2**) are merged into (**A3**). (**B**): time-course of cells stimulated with 0.625 mM histamine showing a fast (seconds) increase (**B1**–**B4**) of green fluorescence (red scale bar = 100 μm). One experiment is representative of 5. (**C**) Quantitative representation of relative fluorescence (RFU) increases with the time from 0 to 60 s. Mean ± SD, *n* = 5 independent measurements; * *p* < 0.1, ** *p* < 0.01.

Fluo-4 AM, a sensitive probe, is used as an indicator in Ca^2+^ mobilization experiments [56,57]. In the absence of histamine stimulation, Caco-2 cells, loaded with Fluo-4, showed only a few green fluorescent spots under confocal microscopy (Figure 1A1–A3).

The very limited, almost non-fluorescent zone (Figure 1A1) could have been related to the absence of living cells. However, under transmitted light microscopy, a well-defined cellular layer appeared with some cellular clusters (Figure 1A2). Merging Figure 1A1, A2 into A3, with overlap of the two panels (A1 and A2), confirmed that the majority of green spots (Figure 1A1) are associated with clustered cells (Figure 1A2) probably due to the aggregative properties of Caco-2 cells [53]. The loading of Fluo-4 AM was confirmed in the absence of histamine by a basal (very low) fluorescence with only a few fluorescent spots (Figure 1A3). As expected, this basal fluorescence was comparable to that observed at time zero of the cell’s treatment with histamine (Figure 1B1). The cell’s treatment with histamine (0.625 mM) resulted in a time-dependent increase in the number of green fluorescent spots, indicating a fast activation (less than 1 min) of Ca^2+^ flow to the cytosol occurred in the cells at histamine signaling (Figure 1B1–B4).

Fluorescence quantification derived from Figure 1A is shown in Figure 1C: a fast increase in intracellular free Ca^2+^ content was noticed at about 40–50 s (s) and became significant at 60 s following histamine stimulation. The rapid increase in fluorescence followed by a sustained plateau (Figure 1B) is consistent with intracellular Ca^2+^ release from endoplasmic reticulum stores and subsequent stabilization through homeostatic mechanisms.

**Figure 2:**
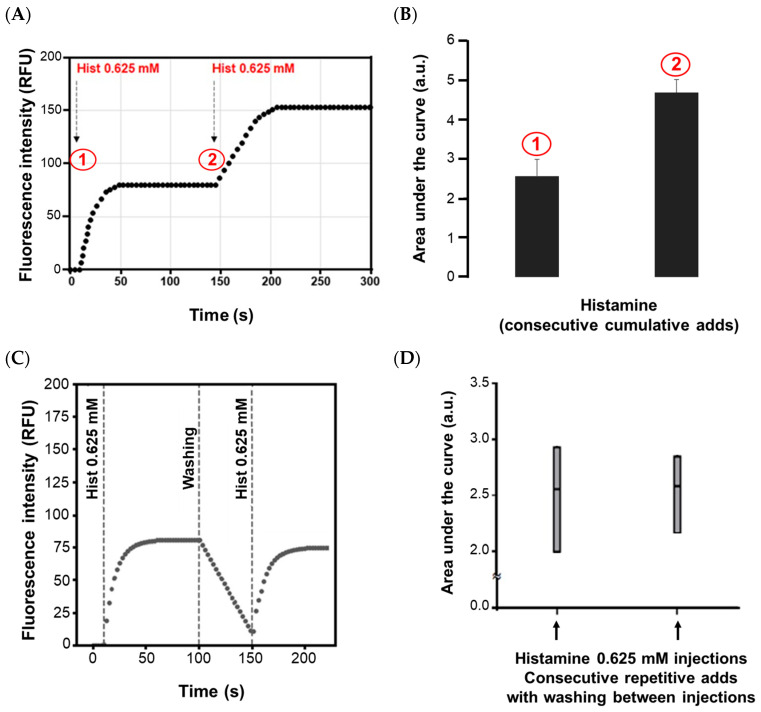
Histamine stimulations: cumulative (**A**,**B**) and repetitive with alternated washings (**C**,**D**). (**A**) Time-course of Fluo-4 fluorescence upon 0.625 mM histamine subsequent cumulative treatments (one experiment representative of 5). (**B**) Quantitative analysis of Fluo-4 fluorescence as cumulative area under the curve (AUC) at 0.625 mM histamine stimulations (mean ± SD, *n* = 5 independent measurements, *p* > 0.01). (**C**) Time-course of Fluo-4 fluorescence upon 0.625 mM histamine subsequent repetitive treatments alternated with washings (PBS 1×, pH 7.2). (**D**) Quantitative analysis of Fluo-4 fluorescence as area under the curve (AUC) at repeated 0.625 mM histamine stimulation after intermediate washing (mean ± SD, *n* = 5 independent measurements, *p* > 0.01).

Sequential applications of histamine 0.625 mM were used to evaluate the reproducibility and kinetics of the fluorescence response. Figure 2A shows the temporal fluorescence profile during consecutive applications (first and second) of 0.625 mM of histamine, while Figure 2B shows quantification of the cumulative fluorescence responses to histamine stimulation (1 and 2). Importantly, Figure 2C,D showed that removal of extracellular histamine by washing leads to a marked decrease in fluorescence toward baseline levels. Subsequent re-stimulation with histamine induces a response of comparable amplitude, indicating that the signal is reversible and not due to cumulative artifacts. Together, these results confirm that the increase in fluorescence is specifically driven by extracellular histamine and reflects a reproducible, receptor-mediated Ca^2+^ response.

The concentration of 0.625 mM histamine used on Caco-2 cells to induce Ca^2+^ mo-bility appears higher compared to commonly used concentrations in histamine-induced signaling studies in other experimental models. For instance, in a previous ex vivo study (murine distal colon muscle) on DAO alleviating the histamine-induced intestinal contraction, the histamine concentration to trigger intestinal motility was much lower (50 μM) [14]. In the current in vitro experimental conditions on intestinal Caco-2 cultured cells, such concentrations did not generate a sufficiently robust and reproducible intracellular Ca^2+^ answer for quantitative analysis. Therefore, a higher concentration (0.625 mM) was retained to obtain consistent and measurable responses above baseline noise. This histamine concentration is comparable with that used in a number of other studies on Caco-2 cells that elicit responses to histamine at relatively high concentrations (1 mM) and with a lethal concentration (LC_50_) of ≅ 10 mM following 24 h of exposure [58]. These results showed that washing steps restore baseline fluorescence. Removal of extracellular histamine resulted in a decrease in fluorescence toward baseline levels. A second histamine application induced a response comparable to the previous one, indicating the signal reversibility.

**Figure 3:**
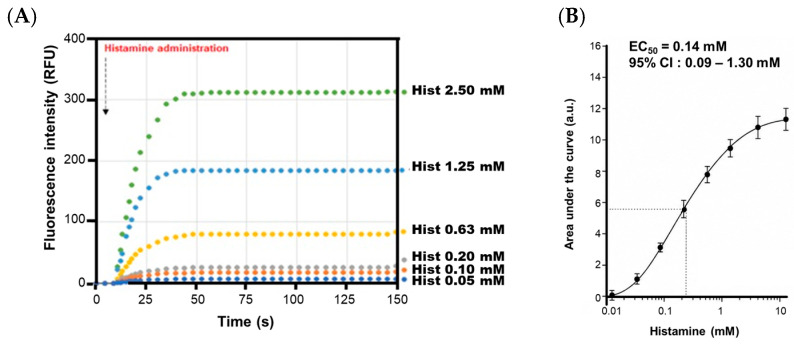
Effect of histamine on Ca^2+^ mobility in 21-day differentiated Caco-2 cells loaded with Fluo-4 AM. (**A**) Kinetics of Fluo-4 fluorescence upon treatment with histamine at different concentrations. For each concentration, one experiment representative of 5 is presented. (**B**) Effect of histamine concentration on the Fluo-4 fluorescence expressed as AUC after subtraction of control values. (Mean ± SD; *n* = 5 independent measurements.)

The fluorescence intensity associated with intracellular Ca^2+^ mobilization increased in a concentration-dependent manner following histamine stimulation (Figure 3A). Nonlinear regression analysis yielded a half effective concentration (EC_50_) of 0.140 mM (95% confidence interval (CI), 0.09–1.30 mM) (Figure 3B). These results indicate that histamine induces intracellular Ca^2+^ signaling in Caco-2 cells within the high micromolar to low millimolar concentration range. Accordingly, a histamine concentration of 1.25 mM histamine was selected for the _V_DAO and Des assays to ensure a robust and reproducible cellular response. Notably, this concentration falls within the upper limit of the EC_50_ 95% CI, providing additional statistical support for its experimental use.

Histamine as a multifactorial bioactive agent, when in excess (from food or gut bacteria) or when intestinal DAO is impaired, may exert deleterious effects locally and via diffusion into the bloodstream. Histamine promotes inflammation by attracting immune cells (mast cells, neutrophils) and may trigger abdominal pain and diarrhea by activating visceral neurons [6,59]. Histamine, as an inflammatory mediator, modulates the increase in epithelial permeability [60] by disrupting the tight junctions between intestinal cells. Thus, histamine will diffuse into the bloodstream causing systemic effects, including pseudo-allergy phenomena. This process can be prevented by orally administering DAO.

### 3.2. _V_DAO and Des Prevented Ca^2+^ Mobility Stimulated by Histamine

**Figure 4:**
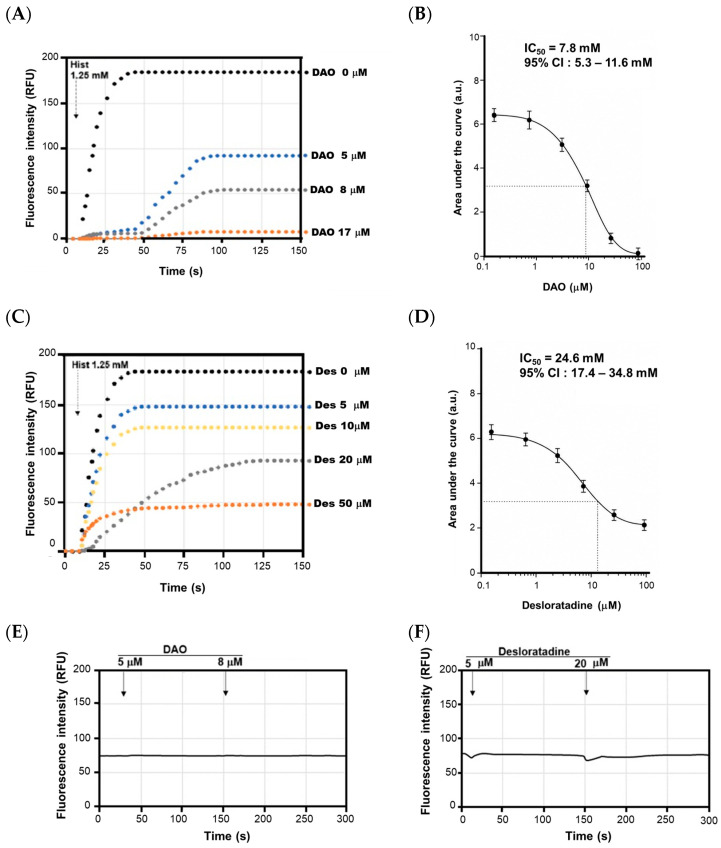
Effect of vDAO and of Des on histamine-induced Ca^2+^ mobilization in 21 days-differentiated Caco-2 cells loaded with Fluo-4. (**A**) Time-course of Fluo-4 fluorescence at Ca^2+^ mobilization triggered by 1.25 mM histamine, under treatment with vDAO at various concentrations (each experiment is representative of 5); (**B**) quantitative analysis of Fluo-4 fluorescence decrease at treatment with different concentrations of vDAO, expressed as AUC (mean ± SD, *n* = 5 independent experiments); (**C**) time-course of Fluo-4 fluorescence at Ca^2+^ mobilization triggered by 1.25 mM histamine at treatment with desloratadine (Des) at various concentrations (each experiment is representative of 5); (**D**) quantitative analysis of Fluo-4 fluorescence decrease at treatment with different concentrations of Des, expressed as AUC (mean ± SD, *n* = 5 independent experiments); (**E**) effect of vDAO at various concentrations on Fluo-4 fluorescence when cells were not exposed to histamine (one experiment representative of 5); (**F**) effect of Des at various concentrations on Fluo-4 fluorescence when cells were not exposed to histamine (one experiment representative of 5).

An endogenous DAO produced by Caco-2 cells has previously been reported [28,58,61,62]. In the current study, all data were recorded considering control conditions in the absence of exogenous vDAO or of Des. Thus, the observed responses in Ca^2+^ mobility stimulated by histamine were ascribed to vDAO or to treatment of cells with Des. A fixed concentration (1.25 mM) of histamine was selected for the subsequent experiments to evaluate the effect of vDAO on Ca^2+^ mobility. At this concentration, histamine showed a low toxicity (about 78–80% viability on cultured Caco-2 cells). The vDAO was found to generate a concentration-dependent protective inhibition of Ca^2+^ mobility triggered by 1.25 mM histamine (Figure 4A). For instance, vDAO at a final concentration of 17 μM produced about 96% inhibition of Ca^2+^ mobilization (Figure 4B).

The effect of Des on histamine-induced Ca^2+^ release was also evaluated. At a con-centration of 50 μM Des, the Ca^2+^ flux was 72% inhibited (Figure 4C). These data indicate a stronger inhibitory effect of vDAO compared to Des (Figure 4D). Practically, vDAO presented a higher potency in reduction in Ca^2+^ mobilization, with a Half Maximal Inhibitory Concentration IC_50_ of 7.8 μM, which was three-fold lower compared to the IC_50_ of 24.6 μM for Des (Figure 4B,D). This effect is in agreement with the previous observation of Limon et al. [63] showing a moderate inhibition of intracellular Ca^2+^ flux at increasing concentrations of Des [63,64].

Our data showed that in the absence of histamine, neither vDAO nor Des induced fluorescence of Fluo-4 probe in Caco-2 cells (Figure 4E,F). In this case, the lack of effect of vDAO or of Des on Ca^2+^ mobility can be considered as a negative control.

Similar protective effects were previously found with vDAO on histamine-induced intestinal smooth muscle contractibility [14], but with an ex vivo model measuring motility of distal colon segments (triggered by histamine and alleviated by DAO). The current in vitro study on cultured intestinal Caco-2 cells and confocal microscopy measurements shows the histamine-induced Ca^2+^ flux dynamics counteracted by DAO. This in vitro model for investigating histamine effects in intestinal cells is much more convenient and easier to apply than the ex vivo model, particularly for the aspects related to Ca^2+^ mobility.

The stability of DAO is related to its concentration and to the nature of the environmental fluids [48]. In this way it was crucial to eliminate possible interference and to choose the best experimental conditions. The DMEM is a rich cell culture medium supplemented with antibiotics, with non-essential amino acids and with fetal bovine serum (FBS) which is a main component of eukaryote cell culture media. The FBS collected from bovine fetal blood [65] may contain serum amine oxidases (SAOs) as well as DAO, which are known to be highly expressed in the placenta [66]. Consistent with this possibility, vDAO activity in the presence of FBS was greater than that measured in the control 0.1 M phosphate buffer (pH 7.2) (Figure S1), suggesting the use of FBS-free medium to avoid interference in further experiments.

It was also of interest to evaluate the vDAO activity as a function of increasing concentrations of histamine. An increase in the reaction rate was observed with histamine concentrations ranging from 0.01 to 10 mM and a slight inhibition was measured at a high concentration (100 mM) of histamine, probably due to substrate inhibition (Figure S1).

A noteworthy feature of histamine oxidation by vDAO is the concomitant production of hydrogen peroxide (H_2_O_2_) (Scheme 1). Table S1 provides supplementary data on the effect of vDAO and histamine on production of H_2_O_2_ and on Ca^2+^ mobility (Table S1A) and on the effect of H_2_O_2_ alone (as a control) at the same concentrations, but in the absence of vegetal DAO and of histamine, on calcium mobility (Table S1B). During oxidation of histamine (1.25 mM) with increasing concentrations of vDAO (5, 8, and 17 µM, as shown in Figure 4), increasing concentrations (7.4, 11.9, and 25.3 µM H_2_O_2_) were found (Table S1A). Separately, H_2_O_2_ was added at the same concentrations (as those produced by DAO) as the control (without histamine and without vDAO), measuring the calcium mobility (Table S1B). Despite the addition of H_2_O_2_ (control), calcium mobility remained unchanged at basal levels (Table S1B) as in the blank (no H_2_O_2_ added). These findings indicate that in our conditions of confocal microscopy the concentrations of H_2_O_2_ generated by vegetal DAO were insufficient to induce detectable calcium mobilization. Hydrogen peroxide is recognized as a redox signaling molecule able to, in eukaryotic cells, modulate intracellular calcium homeostasis by activating calcium-dependent signaling pathways [67]. The absence of a calcium response suggests that H_2_O_2_ accumulation may have been limited in our experimental system measuring the Fluo-4 AM fluorescence. A possible explanation can be the presence of endogenous catalase expressed by the differentiated Caco-2 cells. In fact, Baker & Baker (1992) showed an increase in catalase activity during differentiation of Caco-2 cells [68]. Since all our determinations were carried out with differentiated Caco-2 cells (day 21) the expressed catalase activity could explain the lack of response in terms of Ca^2+^ mobility in the presence of H_2_O_2_ alone added as a control. Thus, the modulation of Ca^2+^ mobility appears to be triggered by histamine and counteracted by DAO.

In terms of adverse effects, DAO commercial products are well tolerated and considered as safe, particularly at short-term administration. Most of them are designed as “food supplements” and contain DAO of pig kidney or legume sources.

However, an aspect to be taken into account is that DAO, in the presence of biogenic amines such as histamine, generates hydrogen peroxide (H_2_O_2_) a known damaging prooxidant [58]. When histamine was added to same Caco-2 cell line pretreated with vDAO only, a substantial decrease in viability (at about 40%) was observed. Conversely, when cells were pretreated with both DAO and Catalase, the observed viability was very high [58]. These previous results clearly indicated the damaging role of H_2_O_2_. This is why our proposed formulations for oral administration include DAO and Catalase [27,43]. Catalase not only protects cells and tissues against H_2_O_2_, but also releases oxygen in situ, shifting the reaction equilibrium in favor of the DAO oxidative decomposition of histamine or of other biogenic amines (i.e., tyramine) (Scheme 1). This bi-enzymatic DAO + Catalase formulation differs from the commercial products containing DAO only. The DAO and Catalase may be used long-term as food supplements, and even for prevention against histamine-related colorectal cancer [13].

DAO is very sensitive to acidic environments (i.e., stomach acidity). To prevent gastric degradation, DAO gastro-resistant formulations with adequate excipients such as Carboxymethyl-starch (CM-Starch) were reported [43].

Antihistamines are administered in order to alleviate local damages generated by secretion of histamine from activated mast cells and from neutrophil infiltrating cells [8,69].

The efficiency of DAO may suggest a substantial role as histaminase, but with the caveat that its action is mostly exerted at the intestinal level. It may act also as a support to antihistamine drugs, particularly in conditions of intestinal histaminosis such as food histaminosis or in cases of inhibition of intestinal DAO by the action of some drugs such as metformin [37].

The current in vitro investigation was conducted considering the substantial differences between antihistamine drugs and DAO histaminase activity. Antihistamine agents block receptors but histamine remains in the system with the possibility of acting on other receptors not blocked by the antihistamine drug. Conversely, DAO as a histaminase disrupts the histamine making it inactive. This aspect is important considering that Des acts specifically on the H1 receptor [9,70,71] leaving open the possibility of histamine to act on H2, H3, or H4 receptors or even on H1 receptors not fully blocked.

We believe that by decomposing histamine in the gut, DAO administration appears as a reinforcement but not a replacement of other medications including antihistamine and anti-inflammatory drugs. This study was conducted in vitro only and our data are not sufficient to support DAO as a clinical agent, but may help to clarify the role of Diamine Oxidase (DAO). The present data may contribute to understanding the role that diamine oxidase could play in future clinical development.

## 4. Conclusions

Vegetal DAO almost totally reduced the histamine-induced intracellular Ca^2+^ mobility. This efficiency of vDAO was higher (up to 96% inhibition) than that of Desloratadine (up to 72% inhibition). The DAO enzyme exerted this protective effect at a concentration 3-fold lower that of Des. This aspect is relevant for potential use of vDAO formulated for oral administration to prevent histamine-related dysfunction and reduce Ca^2+^ mobilization, thereby alleviating its effects.

## Data Availability

All data are contained within the article.

## Supplementary Materials

The following supporting information can be downloaded at https://www.mdpi.com/article/10.3390/cells15131175/s1: Figure S1. DAO activity in different cell culture media. It was assayed in vitro with histamine as substrate at various concentrations in DMEM not supplemented (white) or supplemented (gray) with 15% fetal bovine serum (FBS) or in 100 mM sodium phosphate buffer pH 7.2 (black); *n* = 3 independent experiments (mean ± SD); Table S1. The effect of vDAO at various concentrations and of histamine on production of H_2_O_2_ and on Ca^2+^ mobility (A) versus the effect of H_2_O_2_ alone (control, at same concentrations) on Ca^2+^ mobility (B).

## Abbreviations

The following abbreviations are used in this manuscript:

AAP	aminoantipyrine
AUC	area under the curve
BSA	bovine serum albumine
Ca^2+^	calcium ion
Caco-2 cells	colorectal adenocarcinoma cells
CaCl_2_	calcium chloride
CI	confidence interval
DCHBS	sodium 3,5-dichloro-2-hydroxybenzenesulfonate
Des	desloratadine
DMEM	dulbecco’s modified Eagle essential minimum medium
EC_50_	half effective concentration
EDTA	ethylenediaminetetraacetic acid
RFU.	relative fluorescence unit
Fluo-4 AM	fluorophore-4 acetoxymethyl ester
FBS	fetal bovine serum
HR	histamine receptor
HEPES	4-(2-hydroxyethyl)-1-piperazine-ethanesulfonic acid
Hist	histamine
HRP	horseradish peroxidase
HV	high voltage
H_2_O_2_	hydrogen peroxide
IC_50_	half inhibitory concentration
LC_50_	lethal concentration
Na_2_HPO_4_	dibasic sodium phosphate salt
NaH_2_PO_4_	monobasic sodium phosphate salt
SAO	serum amine oxidases
SDS-PAGE	sodium dodecyl-sulfate polyacrylamide gel electrophoresis
vDAO	vegetal diamine oxidase
λ_Exc_	excitation wavelength
λ_Em_	emission wavelength

## References

[1] Patel R.H., Rahimi N. (2026). Biochemistry, Histamine. StatPearls [Internet].

[2] Krell T., Gavira J.A., Velando F., Fernández M., Roca A., Monteagudo-Cascales E., Matilla M.A. (2021). Histamine: A Bacterial Signal Molecule. Int. J. Mol. Sci..

[3] Jutel M., Akdis C. (2007). Histamine as an Immune Modulator in Chronic Inflammatory Responses. Clin. Exp. Allergy.

[4] Barcik W., Wawrzyniak M., Akdis C.A., O’Mahony L. (2017). Immune Regulation by Histamine and Histamine-Secreting Bacteria. Curr. Opin. Immunol..

[5] Durak-Dados A., Michalski M., Osek J. (2020). Histamine and Other Biogenic Amines in Food. J. Vet. Res..

[6] Tamasi J., Balla Z., Csuka D., Kalabay L., Farkas H. (2022). The Missing Link: A Case of Severe Adverse Reaction to Histamine in Food and Beverages. Am. J. Case Rep..

[7] Visciano P., Schirone M. (2022). Update on Biogenic Amines in Fermented and Non-Fermented Beverages. Foods.

[8] Smolinska S., Winiarska E., Globinska A., Jutel M. (2022). Histamine: A Mediator of Intestinal Disorders—A Review. Metabolites.

[9] Xie H., He S.-H. (2005). Roles of Histamine and Its Receptors in Allergic and Inflammatory Bowel Diseases. World J. Gastroenterol..

[10] Zhao Y., Zhang X., Jin H., Chen L., Ji J., Zhang Z. (2022). Histamine Intolerance—A Kind of Pseudoallergic Reaction. Biomolecules.

[11] Dicks L.M. (2022). Gut Bacteria and Neurotransmitters. Microorganisms.

[12] Molina Perelló P., Puntes Rodríguez M., Coimbra Hurtado J., Garrido Sánchez M., Castillo Ocaña M., Martínez Bonifacio D., Carrera Marcolin L., Cuñé Castellana J., Antonijoan Arbós R. (2025). Evaluation of the Safety and Tolerability of Three Single Ascending Doses of Diamine Oxidase (DAO) in Healthy Volunteers: A Randomized Clinical Trial. Int. J. Transl. Med..

[13] Pietrangeli P., Seguella L., Annunziata G., Casano F., Capuano R., Pesce M., De Conno B., Gigli S., Sarnelli G., Pesce M. (2019). Lathyrus Sativus Diamine Oxidase Counteracts Histamine-induced Cell Proliferation, Migration and Pro-angiogenic Mediators Release in Human Colon Adenocarcinoma Cell Line Caco-2. Phytother. Res..

[14] Neree A.T., Soret R., Marcocci L., Pietrangeli P., Pilon N., Mateescu M.A. (2020). Vegetal Diamine Oxidase Alleviates Histamine-Induced Contraction of Colonic Muscles. Sci. Rep..

[15] Schnedl W.J., Enko D. (2021). Histamine Intolerance Originates in the Gut. Nutrients.

[16] Schnedl W.J., Enko D. (2021). Considering Histamine in Functional Gastrointestinal Disorders. Crit. Rev. Food Sci. Nutr..

[17] Borecki M., Jałocha K., Pysz P., Świtała K., Hrapkowicz R., Czernecka A., Erazmus K., Mroczka M., Kuciel J., Tomczak D. (2025). Histamine Intolerance on Diagnosis and Treatment. Med. Sci..

[18] Carthy E., Ellender T. (2021). Histamine, Neuroinflammation and Neurodevelopment: A Review. Front. Neurosci..

[19] Parsons M.E., Ganellin C.R. (2006). Histamine and Its Receptors. Br. J. Pharmacol..

[20] Jutel M., Akdis M., Akdis C. (2009). Histamine, Histamine Receptors and Their Role in Immune Pathology. Clin. Exp. Allergy.

[21] Sander L.E., Lorentz A., Sellge G., Coeffier M., Neipp M., Veres T., Frieling T., Meier P.N., Manns M.P., Bischoff S.C. (2006). Selective Expression of Histamine Receptors H1R, H2R, and H4R, but Not H3R, in the Human Intestinal Tract. Gut.

[22] Schrammel J.C., König M., Frommer M., Andersen K.S., Kirsten M., Seifert R., Neumann D., Schirmer B. (2023). Histamine H1-and H4-Receptor Expression in Human Colon-Derived Cell Lines. Naunyn-Schmiedeberg’s Arch. Pharmacol..

[23] Lipps C., May T., Wirth D. (2014). 2 Cell Lines. Anim. Cell Biotechnol. Biol. Prod..

[24] Natoli M., Leoni B.D., D’Agnano I., Zucco F., Felsani A. (2012). Good Caco-2 Cell Culture Practices. Toxicol. Vitr..

[25] Fischer L. (2022). Recent Advances in the Application of Microbial Diamine Oxidases and Other Histamine-Oxidizing Enzymes. World J. Microbiol. Biotechnol..

[26] Mondovì B., Riccio P., Agostinelli E., Marcozzi G. (2021). Oxidation of Diamines and Polyamines. The Physiology of Polyamines, Volume I.

[27] Mondovi B., A. Fogel W., Federico R., Calinescu C., A. Mateescu M., C. Rosa A., Masini E. (2013). Effects of Amine Oxidases in Allergic and Histamine-Mediated Conditions. Recent Pat. Inflamm. Allergy Drug Discov..

[28] D’Agostino L., Daniele B., Pignata S., Gentile R., Tagliaferri P., Contegiacomo A., Silvestro G., Polistina C., Bianco A.R., Mazzacca G. (1989). Ornithine Decarboxylase and Diamine Oxidase in Human Colon Carcinoma Cell Line CaCo-2 in Culture. Gastroenterology.

[29] Costa-Catala J., Pellicer-Roca S., Iduriaga-Platero I., Sánchez-Pérez S., Veciana-Nogués M.T., Latorre-Moratalla M.L., Vidal-Carou M.C., Comas-Basté O. (2024). Impact of Technological Factors on Diamine Oxidase (DAO) Activity in Porcine Kidney Extracts as Active Ingredient for the Dietary Management of Histamine Intolerance. Appl. Food Res..

[30] Galvez-Martin P., Gallego E., Solano-Romaní L., Martínez-Puig D., Cabañas J., Castells G., Velasco J. (2023). In Vitro Characterization, Evaluation of Enzymatic Activity and Capacity of Intestinal Absorption of Daogest (Dao Extract from Pig Kidney). Clin. Nutr. ESPEN.

[31] Kettner L., Seitl I., Fischer L. (2020). Evaluation of Porcine Diamine Oxidase for the Conversion of Histamine in Food-relevant Amounts. J. Food Sci..

[32] Arib L., Kaci A. (2021). Diamine Oxidase Use as an Anti-Inflammatory Dietary Supplement.

[33] Costa-Catala J., Kettner L., Latorre-Moratalla M.L., Vidal-Carou M.C., Comas-Basté O., Fischer L. (2025). Comparative Characterization of Diamine Oxidase (DAO) Activity from Plant, Animal and Microbial Matrices. Food Biosci..

[34] Sánchez-Pérez S., Comas-Basté O., Duelo A., Veciana-Nogués M.T., Berlanga M., Latorre-Moratalla M.L., Vidal-Carou M.C. (2022). Intestinal Dysbiosis in Patients with Histamine Intolerance. Nutrients.

[35] Duelo A., Comas-Basté O., Sánchez-Pérez S., Veciana-Nogués M.T., Ruiz-Casares E., Vidal-Carou M.C., Latorre-Moratalla M.L. (2024). Pilot Study on the Prevalence of Diamine Oxidase Gene Variants in Patients with Symptoms of Histamine Intolerance. Nutrients.

[36] Leitner R., Zoernpfenning E., Missbichler A. (2014). Evaluation of the Inhibitory Effect of Various Drugs/Active Ingredients on the Activity of Human Diamine Oxidase In Vitro. Clin. Transl. Allergy.

[37] Yee S.W., Lin L., Merski M., Keiser M.J., Gupta A., Zhang Y., Chien H.-C., Shoichet B.K., Giacomini K.M. (2015). Prediction and Validation of Enzyme and Transporter Off-Targets for Metformin. J. Pharmacokinet. Pharmacodyn..

[38] Hungerford J.M. (2010). Scombroid Poisoning: A Review. Toxicon.

[39] Eraky A.M., Wright A., McDonald D. (2023). Pseudo-Allergies in the Emergency Department: A Common Misdiagnosis of Hypersensitivity Type 1 Allergic Reaction. Cureus.

[40] Parish W. (1983). Intolerance and Allergy to Foods and Food Additives: Its Relevance to Toxicology. Toxic Hazards in Food.

[41] Duelo A., Sánchez-Pérez S., Pellicer-Roca S., Sánchez-Buxens S., Comas-Basté O., Latorre-Moratalla M.L., Vidal-Carou M.C. (2025). Improvement of Histamine Intolerance Symptoms in Pregnant Women with Diamine Oxidase Deficiency: An Exploratory Study. J. Clin. Med..

[42] Tan Z., Ou Y., Cai W., Zheng Y., Li H., Mao Y., Zhou S., Tu J. (2022). Advances in the Clinical Application of Histamine and Diamine Oxidase (DAO) Activity: A Review. Catalysts.

[43] Calinescu C., Mondovi B., Federico R., Ispas-Szabo P., Mateescu M.A. (2012). Carboxymethyl Starch: Chitosan Monolithic Matrices Containing Diamine Oxidase and Catalase for Intestinal Delivery. Int. J. Pharm..

[44] Chantret I., Barbat A., Dussaulx E., Brattain M.G., Zweibaum A. (1988). Epithelial Polarity, Villin Expression, and Enterocytic Differentiation of Cultured Human Colon Carcinoma Cells: A Survey of Twenty Cell Lines. Cancer Res..

[45] Kounga P.C., Neree A.T., Pietrangeli P., Marcocci L., Mateescu M.A. (2022). Faster and Sensitive Zymographic Detection of Oxidases Generating Hydrogen Peroxide. The Case of Diamine Oxidase. Anal. Biochem..

[46] Ahmadifar S., Le T.C., Marcocci L., Pietrangeli P., Mateescu M.A. (2017). Zymographic Approach to Determine the Intrinsic Enzyme Specific Activity of Diamine Oxidase in Presence of Interfering Enzymes. Anal. Chim. Acta.

[47] Calinescu C., Federico R., Mondovi B., Mateescu M.A. (2010). Zymographic Assay of Plant Diamine Oxidase on Entrapped Peroxidase Polyacrylamide Gel Electrophoresis. A Study of Stability to Proteolysis. Anal. Bioanal. Chem..

[48] Neree A.T., Pietrangeli P., Szabo P.I., Mateescu M.A., Marcocci L. (2018). Stability of Vegetal Diamine Oxidase in Simulated Intestinal Media: Protective Role of Cholic Acids. J. Agric. Food Chem..

[49] Bradford M.M. (1976). A Rapid and Sensitive Method for the Quantitation of Microgram Quantities of Protein Utilizing the Principle of Protein-Dye Binding. Anal. Biochem..

[50] Jumarie C., Malo C. (1991). Caco-2 Cells Cultured in Serum-free Medium as a Model for the Study of Enterocytic Differentiation In Vitro. J. Cell. Physiol..

[51] Gee K.R., Brown K., Chen W.U., Bishop-Stewart J., Gray D., Johnson I. (2000). Chemical and Physiological Characterization of Fluo-4 Ca^2+^-Indicator Dyes. Cell Calcium.

[52] Lock J.T., Parker I., Smith I.F. (2015). A Comparison of Fluorescent Ca^2+^ Indicators for Imaging Local Ca^2+^ Signals in Cultured Cells. Cell Calcium.

[53] Meunier V., Bourrie M., Berger Y., Fabre G. (1995). The Human Intestinal Epithelial Cell Line Caco-2; Pharmacological and Pharmacokinetic Applications. Cell Biol. Toxicol..

[54] Miret S., Abrahamse L., de Groene E.M. (2004). Comparison of In Vitro Models for the Prediction of Compound Absorption across the Human Intestinal Mucosa. J. Biomol. Screen..

[55] Thangam E.B., Jemima E.A., Singh H., Baig M.S., Khan M., Mathias C.B., Church M.K., Saluja R. (2018). The Role of Histamine and Histamine Receptors in Mast Cell-Mediated Allergy and Inflammation: The Hunt for New Therapeutic Targets. Front. Immunol..

[56] Persechini A., Lynch J.A., Romoser V.A. (1997). Novel Fluorescent Indicator Proteins for Monitoring Free Intracellular Ca^2+^. Cell Calcium.

[57] Palmer A.E., Tsien R.Y. (2006). Measuring Calcium Signaling Using Genetically Targetable Fluorescent Indicators. Nat. Protoc..

[58] Jumarie C., Séïde M., Marcocci L., Pietrangeli P., Mateescu M.A. (2017). Diamine Oxidase from White Pea (Lathyrus Sativus) Combined with Catalase Protects the Human Intestinal Caco-2 Cell Line from Histamine Damage. Appl. Biochem. Biotechnol..

[59] Siraj H.M., Usaid M., Shaikh S., Dalain M., Ansari Y., Naqvi S.Z., Hossain R., Anum H., Ravi C.A., Pant N. (2025). Bacterial Histamine as a Therapeutic Target for Abdominal Pain in Irritable Bowel Syndrome: A Literature Review. Cureus.

[60] Bischoff S.C., Barbara G., Buurman W., Ockhuizen T., Schulzke J.-D., Serino M., Tilg H., Watson A., Wells J.M. (2014). Intestinal Permeability–a New Target for Disease Prevention and Therapy. BMC Gastroenterol..

[61] Daniele B., Quaroni A. (1991). Effects of Epidermal Growth Factor on Diamine Oxidase Expression and Cell Growth in Caco-2 Cells. Am. J. Physiol.-Gastrointest. Liver Physiol..

[62] Daniele B., Quaroni A. (1990). Polarized Secretion of Diamine Oxidase by Intestinal Epithelial Cells and Its Stimulation by Heparin. Gastroenterology.

[63] Limon L., Kockler D.R. (2003). Desloratadine: A Nonsedating Antihistamine. Ann. Pharmacother..

[64] Berger W.E. (2005). The Safety and Efficacy of Desloratadine for the Management of Allergic Disease. Drug Saf..

[65] Van der Valk J., Mellor D., Brands R., Fischer R., Gruber F., Gstraunthaler G., Hellebrekers L., Hyllner J., Jonker F., Prieto P. (2004). The Humane Collection of Fetal Bovine Serum and Possibilities for Serum-Free Cell and Tissue Culture. Toxicol. Vitr..

[66] Maintz L., Schwarzer V., Bieber T., van der Ven K., Novak N. (2008). Effects of Histamine and Diamine Oxidase Activities on Pregnancy: A Critical Review. Hum. Reprod. Update.

[67] Stone J.R., Yang S. (2006). Hydrogen Peroxide: A Signaling Messenger. Antioxid. Redox Signal..

[68] Baker S.S., Baker R.D. (1992). Antioxidant Enzymes in the Differentiated Caco-2 Cell Line. Vitr. Cell. Dev. Biol.-Anim..

[69] Fogel W., Lewiński A., Jochem J. (2005). Histamine in Idiopathic Inflammatory Bowel Diseases--Not a Standby Player. Folia Medica Cracoviensia.

[70] Binkhorst L.C., Josimovic I., De Vetten D., Nijman T.J., Hauwert N.J., Ahmad S., Van Linden O.P., De Esch I.J., Vischer H.F., Wijtmans M. (2025). Design and Synthesis of Photoswitchable Desloratadine Ligands for Histamine H 1 Receptor Photopharmacology. RSC Med. Chem..

[71] Lu J., Zhao X., Ruan Y., Liu X., Di X., Xu R., Wang J., Qian M., Jin H., Li W. (2024). Desloratadine Ameliorates Paclitaxel-Induced Peripheral Neuropathy and Hypersensitivity Reactions in Mice. Acta Pharmacol. Sin..

