# Supplementary material for: Plant-Derived Diamine Oxidase Modulation of Histamine-Induced Ca2+ Release in Intestinal Caco-2 Cells: A Cellular System to Evaluate Its Histaminase Efficacy
Source: Cells. 2026 Jun 28;15(13):1175. doi: 10.3390/cells15131175 (PMC13359489; doi:10.3390/cells15131175)
Supplement: Supplementary file 1 [file cells-15-01175-s001.zip › cells-4206466-supplementary.pdf]

Supplementary informations

Plant-derived Diamine oxidase modulation of histamine-induced Ca^{2+} release in intestinal Caco-2 cells: a cellular system to evaluate its histaminase efficacy

Armelle Tchoumi Neree [1-2], Catherine Jumarie [3], Lucia Marcocci [4], Paola Pietrangeli [4] and Mircea Alexandru Mateescu [1-2*]

1 Department of Chemistry, Université du Quebec à Montreal, Montreal (QC) Canada, H3C 3P8; tchoumi_neree.armelle@courrier.uqam.ca and mateescu.m-alexandru@uqam.ca
2 Centre Protéo – UQAM, Université du Quebec à Montreal, Montreal (QC) Canada, H3C 3P8
3 Department of Biological Sciences, Groupe de recherche du TOXEN - UQAM, Université du Quebec à Montreal, Montreal (QC) Canada, H3C 3P8; jumarie.catherine@uqam.ca
4 Department of Biochemical Sciences "A. Rossi Fanelli", "Sapienza" University of Rome 1, Rome, Italy, 00185; lucia.marcocci@uniroma1.it and paola.pietrangeli@uniroma1.it
* Correspondence: mateescu.m-alexandru@uqam.ca ; Tel.: (+1)514-987 4319

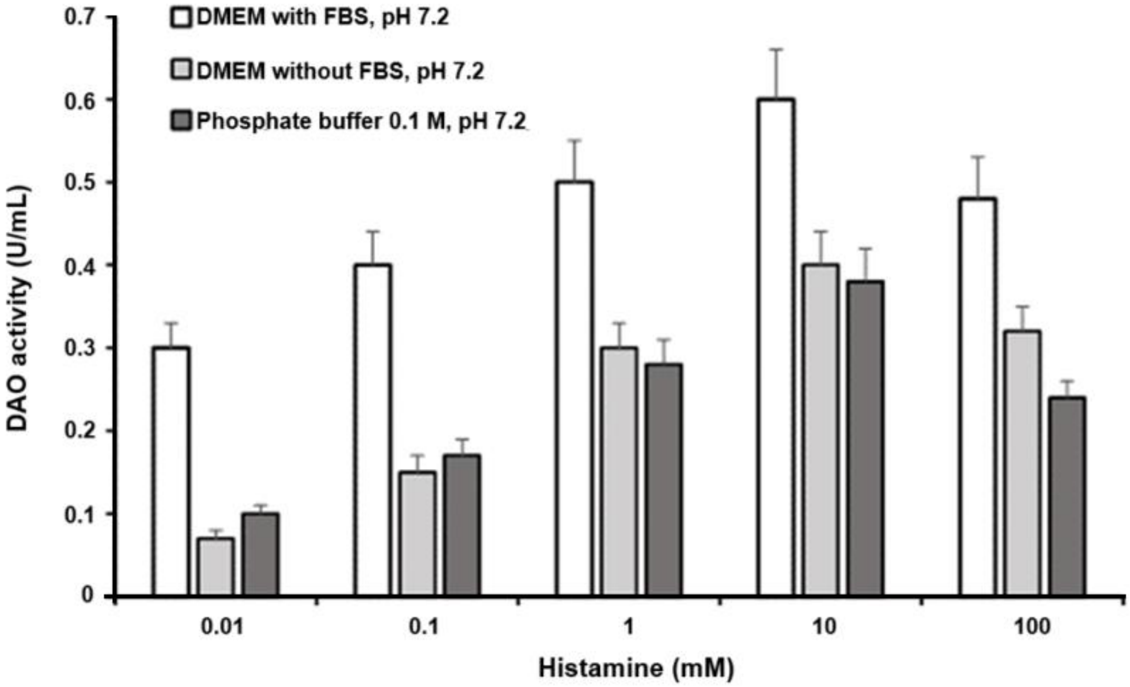

Figure S1 – DAO activity in different cell culture media. It was assayed in vitro with histamine as substrate at various concentrations in DMEM not supplemented (white) or supplemented (grey) with 15% fetal bovine serum (FBS) or in 100 mM sodium phosphate buffer pH 7.2 (black); n=3 independent experiments (mean ± SD).

Table S1 – The effect of vDAO at various concentrations and of histamine on production of H_2O_2 and on Ca^{2+} mobility (**A**) versus the effect of H_2O_2 alone (control, at same concentrations) on Ca^{2+} mobility (**B**).

A)

vDAO (μM)	Histamine (mM)	**H_2O_2 produced (μM)**	**RFU**
0	1.25	0	190 ± 21
5		7.43	90 ± 7
8		11.89	50 ± 2
17		25.27	5 ± 1

B)

**H_2O_2 added to the cell-culture medium (μM)**	**RFU**
0	9 ± 3
7.43	8 ± 2
11.89	9 ± 2
25.27	10 ± 2
